# Mimicking the Lipid Peroxidation Inhibitory Activity of Phospholipid Hydroperoxide Glutathione Peroxidase (GPx4) by Using Fatty Acid Conjugates of a Water-Soluble Selenolane

**DOI:** 10.3390/molecules200712364

**Published:** 2015-07-07

**Authors:** Michio Iwaoka, Arisa Katakura, Jun Mishima, Yoshimi Ishihara, Amit Kunwar, Kavirayani Indira Priyadarsini

**Affiliations:** 1 Department of Chemistry, School of Science, Tokai University, Kitakaname, Hiratsuka-shi, Kanagawa 259-1292, Japan; E-Mails: puing1103@gmail.com (A.K.); j-mishima@kofu.tokai.ed.jp (J.M.); ishihara@tokai-u.jp (Y.I.); 2 Radiation and Photochemistry Division, Bhabha Atomic Research Centre, Trombay, Mumbai 400085, India; E-Mails: kamit@barc.gov.in (A.K.); kindira@barc.gov.in (K.I.P.)

**Keywords:** antioxidant, lipid peroxidation, TBARS, colony formation assay

## Abstract

A series of fatty acid conjugates of *trans*-3,4-dihydroxy-1-selenolane (DHS) were synthesized by reacting DHS with appropriate acid chlorides. The obtained monoesters were evaluated for their antioxidant capacities by the lipid peroxidation assay using a lecithin/cholesterol liposome as a model system. The observed antioxidant capacities against accumulation of the lipid hydroperoxide (LOOH) increased with increasing the alkyl chain length and became saturated for dodecanoic acid (C_12_) or higher fatty acid monoesters, for which the capacities were much greater than those of DHS, its tridecanoic acid (C_13_) diester, and PhSeSePh. On the other hand, the bacteriostatic activity of myristic acid (C_14_) monoester, evaluated through the colony formation assay using *Bacillus subtilis*, indicated that it has higher affinity to bacterial cell membranes than parent DHS. Since DHS-fatty acid conjugates would inhibit lipid peroxidation through glutathione peroxidase (GPx)-like 2e^−^ mechanism, higher fatty acid monoesters of DHS can mimic the function of GPx4, which interacts with LOOH to reduce it to harmless alcohol (LOH). Importance of the balance between hydrophilicity and lipophilicity for the design of effective GPx4 mimics was suggested.

## 1. Introduction

**Scheme 1:**
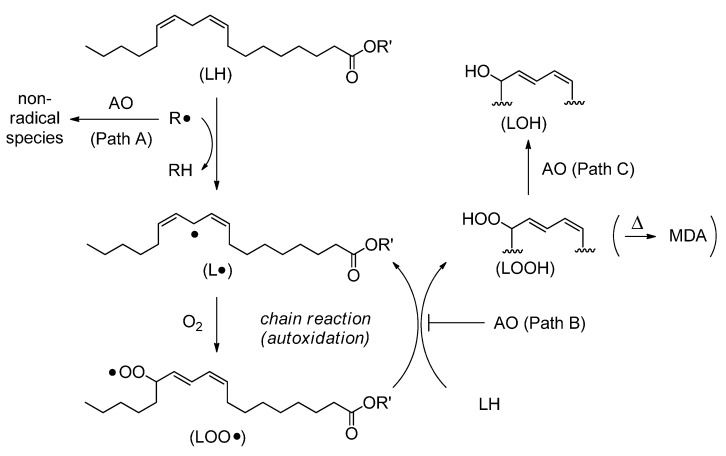
Possible functions of an antioxidant (AO) to inhibit lipid peroxidation induced by a free radical (R•). MDA is malondialdehyde.

Phospholipid hydroperoxide glutathione peroxidase (GPx4) [1,2] is a unique member of a glutathione peroxidase family as it exists in a monomeric form and catalyzes the reduction of phospholipid hydroperoxide (LOOH) with water-soluble thiol substrates, such as glutathione (GSH). This antioxidant enzyme incorporates a selenocysteine residue at the redox active center like typical cytosolic glutathione peroxidase (GPx1), which forms a tetrameric structure and catalyzes the reduction of hydrogen peroxide (H_2_O_2_). The importance of GPx4 in pathophysiology has been well documented in relation to the structure and function of spermatozoa [3], as well as embryogenesis and apoptosis of cells [4,5]. Although a number of organoselenium compounds, most of which are aromatic diselenides and their equivalents, have already been examined as mimics of GPx1 [6,7,8], those which specifically interact with LOOH, like GPx4, to reduce it to harmless alcohol (LOH) have seldom been pursued. In literature, several lipophilic organoselenium compounds, such as Trolox derivatives [9] and α-selenotocopherol [10], were reported to inhibit accumulation of LOOH (Scheme 1). However, their roles as antioxidants (AOs) were most likely explained by the radical scavenging 1e^−^ (path A or B) rather than the GPx4-like peroxidase (2e^−^) mechanism (path C).

We previously reported that *trans*-3,4-dihydroxy-1-selenolane (DHS, **1**), a water-soluble cyclic selenide, has unique redox properties as a GPx1 mimic [11]. Its derivatives were recently synthesized by reacting **1** with various acid chlorides or alkyl halides [12,13], and their antioxidant capacities were evaluated based on the lipid peroxidation assay using a liposome [12]. As a result, it was suggested that the combination of a hydrophilic selenide moiety (*i.e.*, **1**) as a redox center with a higher fatty acid as a lipophilic unit is an effective approach to a new class of AOs that inhibit accumulation of LOOH through a 2e^−^ mechanism (path C in Scheme 1). The maximal antioxidant capacity was indeed suggested when myristic acid (C_14_) was coupled with **1**. Incorporation of the DHS-fatty acid conjugate into the liposome was also evidenced [12]. However, the exact chain-length effect on the capacity, as well as the interaction with living cell membranes, has not yet been well elucidated. In this context, we carried out herein comprehensive analysis of the effect of the alkyl chain length on the GPx4-like antioxidant activity by employing a series of DHS-fatty acid conjugates (**2a**–**l**) based on the lipid peroxidation assay using a lecithin/cholesterol liposome. Their affinity to living cell membranes was interpreted by evaluating the bacteriostatic activity of C_14_ monoester **2h** through the standard colony formation assay using *Bacillus subtilis*. The results are discussed in light of the possible GPx4-like function of **2**.

## 2. Results and Discussion

### 2.1. Synthesis of DHS-Fatty Acid Conjugates **2**

**Scheme 2:**
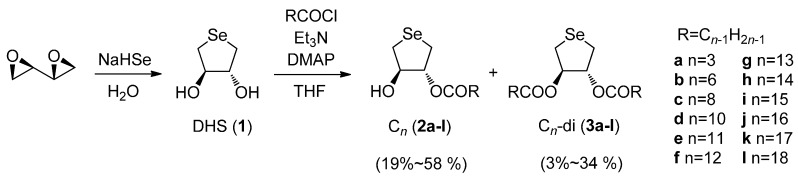
Synthesis of DHS-fatty acid conjugates **2a**–**l** and **3a**–**l**.

According to Scheme 2, a series of fatty acid monoesters of **1** (compounds **2a**–**l**) with an alkyl chain of differing length were synthesized. DHS (**1**), obtained from 1,3-butadiene diepoxide by the reaction with sodium hydrogen selenide (NaHSe) in water [14], was treated with various acid chlorides (RCOCl) in the presence of organic bases to afford the corresponding monoesters **2a**–**l** in moderate yields. From the reaction mixture, diesters **3a**–**l** were also isolated as minor products. The structures of these DHS-fatty acid conjugates were unambiguously characterized by ^1^H-, ^13^C-, and ^77^Se-NMR and elementary analyses (see the experimental section). In the following sections, monoesters **2a**–**l** and diester **3g** were employed for evaluation of their antioxidant capacities to inhibit lipid peroxidation, and parent **1** and C_14_ monoester **2h** were employed for the colony formation assay using *Bacillus subtilis* to elucidate their affinity to the cell membrane. In the both assays, diphenyl diselenide (PhSeSePh, **4**), for which the antioxidant activities on various cell lines have been studied [15,16,17,18], was selected as a reference compound.

### 2.2. Lipid Peroxidation Assay Using Lecithin/Cholesterol Liposome

**Figure 1:**
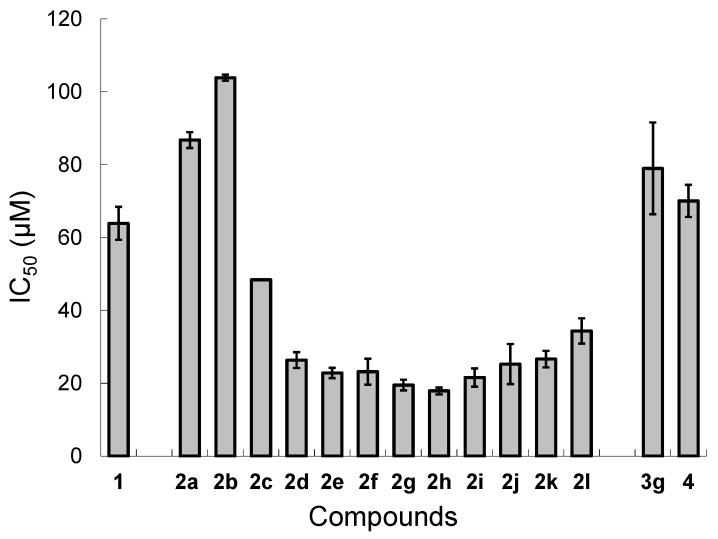
50% inhibitory concentrations (IC_50_) for DHS (**1**), C_3_ (**2a**), C_6_ (**2b**), C_8_ (**2c**), C_10_ (**2d**), C_11_ (**2e**), C_12_ (**2f**), C_13_ (**2g**), C_14_ (**2h**), C_15_ (**2i**), C_16_ (**2j**), C_17_ (**2k**), C_18_ (**2l**), C_13_-di (**3g**), and PhSeSePh (**4**) against lipid peroxidation of soybean lecithin/cholesterol liposome. AAPH (3.1 mM) was used as a hydrophilic radical initiator. The assay conditions were at pH 7.4 and 37 °C for 3 h. Error bars are given as standard deviations. The data for **1**, **2a**, **2f**, **2h**, **2j**, **2l**, and **4** were quoted from Ref. [12]. Copyright Wiley-VCH Verlag GmbH & Co. KGaA. Reproduced with permission.

Lipid peroxidation experiments were carried out following the previous method [12]. A solution of lecithin/cholesterol liposome at pH 7.4 containing 0–120 μM AO was added with hydrophilic 2,2′-azobis(2-amidinopropane) dihydrochloride (AAPH) to initiate lipid peroxidation. The mixture was incubated at 37 °C for 3 h. The amount of LOOH produced was then determined by the thiobarbituric acid reactive substance (TBARS) method [19,20]. The amounts of the MDA-TBA adduct produced were standardized by using the amount obtained in the absence of AO as a positive control and that obtained without addition of AAPH as a negative control. The 50% inhibitory concentrations (IC_50_) obtained for **1**, **2a**–**l**, **3g**, and **4** are graphically shown in Figure 1.

Compared with the parent selenolane **1** (IC_50_ = 64 μM), the C_3_ and C_6_ monoesters **2a** and **2b**, showed larger IC_50_ values, indicating that these DHS conjugates are less active than **1** as AOs in lipid peroxidation inhibition. Lipid peroxidation inhibitory activities of C_13_ diester (**3g**) and reference **4** were not significantly different from that of **1** based on the standard deviations. On the other hand, DHS-fatty acid conjugates **2c**–**l** with a long alkyl chain (8 ≤ *n* ≤ 18) exhibited better antioxidant activities than **1**. It is interesting to see that the IC_50_ value decreases with the alkyl chain length but it begins to increase when the alky chain is longer than C_17_ (compound **2k**). Consequently, the high antioxidant capacities were observed for C_12_ to C_16_ monoesters **2f**–**j** (IC_50_ = 18–25 μM). The results are in agreement with the trend previously deduced on the basis of the IC_50_ values obtained only for **2a**, **2f**, **2h**, **2j**, and **2l** [12]. Thus, the effect of the alkyl chain length on the antioxidant capacity of DHS-fatty acid conjugates to inhibit lipid peroxidation was undoubtedly confirmed. In addition to the observed unique redox properties, it is also notable that only monoesters of DHS are good AOs and diester **3g** is not more active than **1**. This strongly suggests that the balance between hydrophilicity and lipophilicity is important for the design of AOs that can effectively inhibit accumulation of LOOH.

### 2.3. Colony Formation Assay

**Figure 2:**
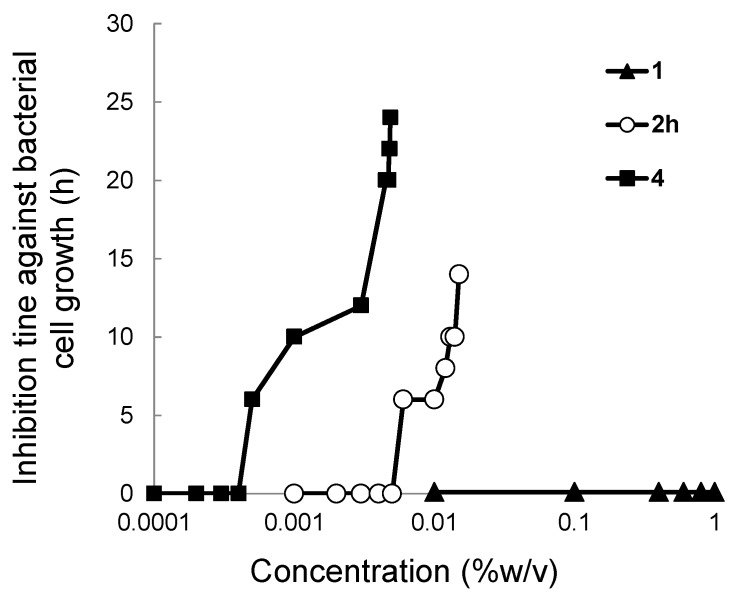
The inhibition time against growth of *Bacillus subtilis* subsp. *subtilis* (NBRC3134) as a function of the concentrations of DHS (**1**), C_14_ (**2h**) and PhSeSePh (**4**).

Subsequently, the bacteriostatic effect of DHS (**1**), C_14_ (**2h**), and PhSeSePh (**4**) against *Bacillus subtilis* subsp. *subtilis* NBRC3134 strain [21,22] was examined by adding these compounds into a broth. In this assay, we selected **2h** as a representative DHS-fatty acid conjugate because **2h** was one of the best AOs in the liposomal assay as described above although other DHS-fatty acid conjugates may have stronger interaction to the bacterial cell membrane. The rate of the bacterial cell growth/multiplication was determined by counting the colony forming units, and the inhibition time against the cell growth was obtained as a function of the concentration of AO (%w/v) added. The results (Figure 2) show that **2h** and **4** inhibit the bacterial growth at the concentrations of >0.006% (>160 μM) and >0.0005% (>16 μM), respectively, whereas **1** did not affect the growth at all at the concentration up to 1% (60 mM). Since the bacteriostatic effect of a chemical/drug depends on its lipophilicity or affinity to the cell membrane [23,24], these results can be interpreted by assuming that **4** strongly interacts with the bacterial cell membrane due to its high hydrophobicity, whereas the interaction of **1** is poor due to its water-solubility. Monoester **2h**, which exhibited the high antioxidant activity in the lipid peroxidation assay using a liposome, showed an intermediate bacteriostatic effect, suggesting its ability to localize in the cell membrane. Although the affinity of **2h** to the bacterial cell membrane was lower than that of **4**, the antioxidant capacity of **2h** was remarkably higher in the lipid peroxidation assays (see Figure 1). These results are also supported by our previous study [12], wherein the ratios of **1** and **2h** incorporated in the lecithin/cholesterol liposome were observed to be 0% and ~50%, respectively. Thus, higher fatty acid conjugates of DHS would easily localize in the membranes.

### 2.4. Antioxidant Function as Possible GPx4 Mimics

**Scheme 3:**
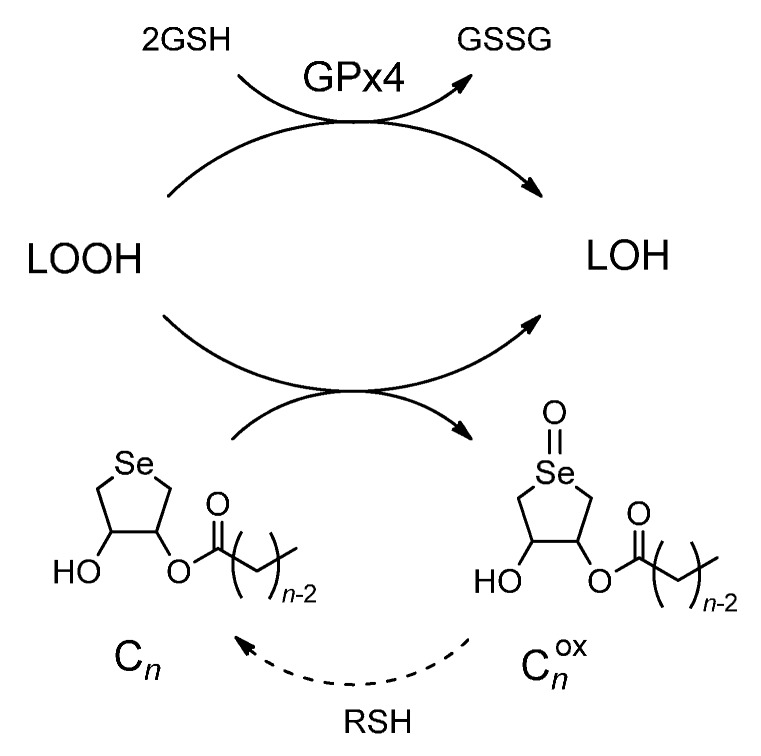
Antioxidant function of DHS-fatty acid conjugates (C*_n_*) as possible GPx4 mimics.

The antioxidant function of lipophilic DHS-fatty acid conjugates **2** in inhibition of lipid peroxidation was previously proposed as follows [12]. The monoesters (C*_n_*) would come into the liposome membrane and effectively interact with LOOH, which is generated as a product of lipid peroxidation induced by AAPH radical (Scheme 1). Through the GPx-like 2e^−^ mechanism, the selenides (C*_n_*) would be oxidized to the corresponding selenoxides (C*_n_*^ox^), releasing LOH as a counter-product (Scheme 3). This reaction mechanism is supported by the results obtained in this study from the lipid peroxidation assay (Figure 1) and the colony formation assay (Figure 2). Thus, it is obvious that the antioxidant function of **2** is similar to that of GPx4, suggesting that higher fatty acid monoesters of DHS can mimic the GPx4 function in terms of the selective reduction of LOOH to LOH through a 2e^−^ mechanism. Produced selenoxides (C*_n_*^ox^) would be easily reduced back to the selenides (C*_n_*) by the reaction with a thiol substrate [12,25]. Therefore, if an appropriate thiol is available, **2** would catalyze degradation of LOOH to LOH. Thus, DHS-fatty acid conjugates **2** may have merits as AOs in practical applications.

## 3. Experimental Section

### 3.1. General Information

DHS (**1**) was synthesized according to the literature method [14]. Tridecanoyl chloride, pentadecanoyl chloride, and heptadecanoyl chloride were synthesized by the reaction of the corresponding acids with thionyl chloride in dichloromethane under reflux conditions. After concentration in vacuo, the resulting crude products were employed for the reaction with **1**. Other reagents were commercially available and used without further purification. ^1^H- (500 MHz), ^13^C- (125.8 MHz), and ^77^Se-NMR (95.4 MHz) spectra were measured in CDCl_3_ at 298 K on a Bruker AV500 NMR spectrometer (Billerica, MA, USA). Gel permeation chromatograph was performed on a JAI LC-918 HPLC system (Tokyo, Japan). Elementary analysis was performed on a Perkin Elmer 2400II elemental analyzer (Waltham, MA, USA).

### 3.2. Synthesis of DHS-Fatty Acid Conjugates **2a**–**l**

To a solution of **1** (114 mg, 0.68 mmol) in THF (5 mL) were added triethylamine (380 μL, 2.73 mmol), *N*,*N*-dimethyl-4-aminopyridine (16.3 mg, 0.13 mmol), and acid chloride (1.1 mmol). The mixture was stirred at room temperature for 4 h. The solution was evaporated, and the residual materials were dissolved in ether. After washing the ethereal layer with saturated aqueous ammonium chloride, sodium bicarbonate, and then sodium chloride solutions, the obtained crude products were purified by silica gel column chromatography (ether-hexane) and gel permeation chromatography (chloroform). The monoester (**2**) was obtained as a major product with a slight amount of the diester (**3**). Synthesis and spectral data for monoesters C_3_ (**2a**), C_12_ (**2f**), C_14_ (**2h**), C_16_ (**2j**), and C_18_ (**2l**) and the corresponding diesters were reported elsewhere [12].

*(±)-trans-4-Hydroxytetrahydroselenophen-3-yl hexanoate* (**2b**): Yield 38%. Colorless oil. ^1^H-NMR (CDCl_3_) δ 0.80 (t, *J* = 7.0 Hz, 3H), 1.20 (m, 4H), 1.50 (m, 2H), 2.22 (t, *J* = 7.6 Hz, 2H), 2.80 (dd, *J* = 2.9, 11.3 Hz, 1H), 2.85 (dd, *J* = 2.9, 10.7 Hz, 1H), 3.04 (dd, *J* = 4.4, 10.7 Hz, 1H), 3.15 (dd, *J* = 4.9, 11.3 Hz, 1H), 3.3 (br, 1H), 4.35 (m, 1H), 5.18 (m, 1H); ^13^C-NMR (CDCl_3_) δ 13.9, 22.2, 24.5, 24.5, 28.0, 31.1, 34.3, 76.8, 80.4, 173.3; ^77^Se-NMR (CDCl_3_) δ 97.4; Anal. Calcd for C_10_H_18_O_3_Se: C, 45.29; H, 6.84. Found: C, 45.09; H, 7.14.

*(±)-trans-Tetrahydroselenophen-3,4-diyl dihexanoate* (**3b**): Yield 18%. Colorless oil. ^1^H-NMR (CDCl_3_) δ 0.82 (t, *J* = 6.9 Hz, 6H), 1.23 (m, 8H), 1.54 (m, 4H), 2.23 (t, *J* = 7.5 Hz, 4H), 2.86 (dd, *J* = 2.7, 10.5 Hz, 2H), 3.13 (dd, *J* = 4.1, 10.9 Hz, 2H), 5.28 (m, 2H); ^13^C-NMR (CDCl_3_) δ 13.8, 22.2, 24.4, 24.5, 31.1, 34.1, 77.8, 172.5; ^77^Se-NMR (CDCl_3_) δ 113.5. The purity was confirmed by the NMR spectra (see Supplementary Materials).

*(±)-trans-4-Hydroxytetrahydroselenophen-3-yl octanoate* (**2c**): Yield 33%. Colorless oil. ^1^H-NMR (CDCl_3_) δ 0.85 (t, *J* = 7.0 Hz, 3H), 1.25 (m, 8H), 1.58 (m, 2H), 2.29 (t, *J* = 7.6 Hz, 2H), 2.64 (d, *J* = 5.5 Hz, 1H), 2.87 (dd, *J* = 3.0, 11.3 Hz, 1H), 2.92 (dd, *J* = 3.0, 10.7 Hz, 1H), 3.12 (dd, *J* = 4.4, 10.7 Hz, 1H), 3.22 (dd, *J* = 5.0, 11.3 Hz, 1H), 4.40 (m, 1H), 5.24 (m, 1H); ^13^C-NMR (CDCl_3_) δ 14.1, 22.6, 24.4, 24.9, 28.2, 28.9, 29.0, 31.6, 34.3, 77.0, 80.3, 173.2; ^77^Se-NMR (CDCl_3_) δ 92.4; Anal. Calcd for C_12_H_22_O_3_Se: C, 49.15; H, 7.56. Found: C, 48.83; H, 7.74.

*(±)-trans-Tetrahydroselenophen-3,4-diyl dioctanoate* (**3c**): Yield 34%. Colorless oil. ^1^H-NMR (CDCl_3_) δ 0.85 (t, *J* = 7.0 Hz, 6H), 1.25 (m, 16H), 1.57 (m, 4H), 2.28 (m, 4H), 2.91 (m, 2H), 3.18 (dd, *J* = 4.3, 11.0 Hz, 2H), 5.33 (m, 2H); ^13^C-NMR (CDCl_3_) δ 14.1, 22.6, 24.3, 24.9, 28.9, 29.0, 31.6, 34.2, 77.9, 172.6; ^77^Se-NMR (CDCl_3_) δ 113.6; Anal. Calcd for C_20_H_36_O_4_Se: C, 57.27; H, 8.65. Found: C, 57.30; H, 8.91.

*(±)-trans-4-Hydroxytetrahydroselenophen-3-yl decanoate* (**2d**): Yield 34%. Colorless oil. ^1^H-NMR (CDCl_3_) δ 0.86 (t, *J* = 6.9 Hz, 3H), 1.26 (m, 12H), 1.58 (m, 2H), 2.29 (t, *J* = 7.6 Hz, 2H), 2.42 (br, 1H), 2.88 (dd, *J* = 3.0, 11.3 Hz, 1H), 2.93 (dd, *J* = 3.0, 10.7 Hz, 1H), 3.13 (dd, *J* = 4.4, 10.7 Hz, 1H), 3.22 (dd, *J* = 5.1, 11.3 Hz, 1H), 4.41 (m, 1H), 5.25 (m, 1H); ^13^C-NMR (CDCl_3_) δ 14.1, 22.7, 24.3, 24.9, 28.2, 29.0, 29.2, 29.2, 29.4, 31.8, 34.3, 77.0, 80.3, 173.2; ^77^Se-NMR (CDCl_3_) δ 91.6; Anal. Calcd for C_14_H_26_O_3_Se: C, 52.33; H, 8.16. Found: C, 52.40; H, 8.50.

*(±)-trans-Tetrahydroselenophen-3,4-diyl didecanoate* (**3d**): Yield 19%. Colorless oil. ^1^H-NMR (CDCl_3_) δ 0.9 (t, *J* = 6.88 Hz, 3H), 1.26 (m, 24H), 1.58 (m, 4H), 2.30 (t, *J* = 7.5 Hz, 4H), 2.94 (dd, *J* = 2.7, 10.3 Hz, 2H), 3.21 (dd, *J* = 4.1, 11.0 Hz, 2H), 5.34 (m, 2H); ^13^C-NMR (CDCl_3_) δ 14.1, 22.7, 24.4, 24.9, 29.1, 29.2, 29.3, 29.4, 31.9, 34.3, 77.9, 172.7; ^77^Se-NMR (CDCl_3_) δ 113.7; Anal. Calcd for C_24_H_44_O_4_Se: C, 60.61; H, 9.33. Found: C, 60.14; H, 9.47.

*(±)-trans-4-Hydroxytetrahydroselenophen-3-yl undecanoate* (**2e**): Yield 37%. Colorless crystals. m.p. 40–42 °C. ^1^H-NMR (CDCl_3_) δ 0.84 (t, *J* = 6.9 Hz, 3H), 1.23 (m, 14H), 1.56 (m, 2H), 2.27 (t, *J* = 7.6 Hz, 2H), 2.8 (br, 1H), 2.86 (dd, *J* = 3.0, 11.3 Hz, 1H), 2.91 (dd, *J* = 3.0, 10.7 Hz, 1H), 3.10 (dd, *J* = 4.4, 10.7 Hz, 1H), 3.21 (dd, *J* = 5.0, 11.3 Hz, 1H), 4.38 (m, 1H), 5.23 (m, 1H); ^13^C-NMR (CDCl_3_) δ 14.1, 22.7, 24.4, 24.9, 28.1, 29.1, 29.2, 29.3, 29.4, 29.5, 31.9, 34.3, 77.0, 80.3, 173.3; ^77^Se-NMR (CDCl_3_) δ 94.0; Anal. Calcd for C_15_H_28_O_3_Se: C, 53.72; H, 8.42. Found: C, 53.88; H, 8.56.

*(±)-trans-Tetrahydroselenophen-3,4-diyl diundecanoate* (**3e**): Yield 11%. Colorless oil. ^1^H-NMR (CDCl_3_) δ 0.86 (t, *J* = 7.0 Hz, 6H), 1.27 (m, 28H), 1.59 (m, 4H), 2.29 (m, 4H), 2.93 (m, 2H), 3.20 (dd, *J* = 4.3, 11.0 Hz, 2H), 5.35 (m, 2H); ^13^C-NMR (CDCl_3_) δ 14.1, 22.7, 24.3, 24.9, 29.1, 29.2, 29.3, 29.5, 29.6, 31.9, 34.3, 77.9, 172.6; ^77^Se-NMR (CDCl_3_) δ 113.6; Anal. Calcd for C_26_H_48_O_4_Se: C, 62.01; H, 9.61. Found: C, 61.85; H, 10.01.

*(±)-trans-4-Hydroxytetrahydroselenophen-3-yl tridecanoate* (**2g**): Yield 43%. Colorless crystals. m.p. 50–52 °C. ^1^H-NMR (CDCl_3_) δ 0.83 (t, *J* = 6.9 Hz, 3H), 1.21 (m, 18H), 1.56 (m, 2H), 2.23 (t, *J* = 7.6 Hz, 2H), 2.85 (dd, *J* = 3.0, 11.3 Hz, 1H), 2.90 (dd, *J* = 3.0, 10.7 Hz, 1H), 3.0 (br, 1H), 3.09 (dd, *J* = 4.4, 10.7 Hz, 1H), 3.26 (dd, *J* = 5.0, 11.3 Hz, 1H), 4.38 (m, 1H), 5.23 (m, 1H); ^13^C-NMR (CDCl_3_) δ 14.1, 22.7, 24.4, 24.9, 28.1, 29.1, 29.2, 29.3, 29.4, 29.6, 29.6, 31.9, 34.3, 76.9, 80.3, 173.3; ^77^Se-NMR (CDCl_3_) δ 94.7; Anal. Calcd for C_17_H_32_O_3_Se: C, 56.19; H, 8.88. Found: C, 56.33; H, 8.98.

*(±)-trans-Tetrahydroselenophen-3,4-diyl ditridecanoate* (**3g**): Yield 10%. Colorless crystals. m.p. 37–39 °C. ^1^H-NMR (CDCl_3_) δ 0.86 (t, *J* = 6.9 Hz, 6H), 1.25 (m, 36H), 1.59 (m, 4H), 2.29 (m, 4H), 2.92 (m, 2H), 3.20 (m, 2H), 5.35 (m, 2H); ^13^C-NMR (CDCl_3_) δ 14.1, 22.7, 24.3, 24.9, 29.1, 29.2, 29.3, 29.5, 29.6, 29.6, 31.9, 34.3, 77.9, 172.6; ^77^Se-NMR (CDCl_3_) δ 113.7; Anal. Calcd for C_30_H_56_O_4_Se: C, 64.37; H, 10.08. Found: C, 64.11; H, 10.41.

*(±)-trans-4-Hydroxytetrahydroselenophen-3-yl pentadecanoate* (**2i**): Yield 29%. Colorless crystals. m.p. 60–62 °C. ^1^H-NMR (CDCl_3_) δ 0.83 (t, *J* = 6.9 Hz, 3H), 1.21 (m, 22H), 1.56 (m, 2H), 2.36 (t, *J* = 7.6 Hz, 2H), 2.85 (dd, *J* = 3.0, 11.3 Hz, 1H), 2.90 (dd, *J* = 3.0, 10.7 Hz, 1H), 3.09 (dd, *J* = 4.4, 10.7 Hz, 1H), 3.19 (dd, *J* = 5.0, 11.3 Hz, 1H), 4.38 (m, 1H), 5.23 (m, 1H); ^13^C-NMR (CDCl_3_) δ 14.1, 22.7, 24.4, 24.9, 28.1, 29.1, 29.2, 29.3, 29.4, 29.6, 29.6, 29.7, 29.7, 31.9, 34.3, 76.9, 80.3, 173.3; ^77^Se-NMR (CDCl_3_) δ 94.9; Anal. Calcd for C_19_H_36_O_3_Se: C, 58.30; H, 9.27. Found: C, 58.48; H, 9.46.

*(±)-trans-Tetrahydroselenophen-3,4-diyl dipentadecanoate* (**3i**): Yield 4%. Colorless crystals. m.p. 49–50 °C. ^1^H-NMR (CDCl_3_) δ 0.89 (t, *J* = 7.0 Hz, 6H), 1.28 (m, 44H), 1.62 (m, 4H), 2.32 (m, 4H), 2.95 (m, 2H), 3.23 (m, 2H), 5.38 (m, 2H); ^13^C-NMR (CDCl_3_) δ 14.1, 22.7, 24.3, 24.9, 29.1, 29.2, 29.4, 29.5, 29.6, 29.7, 29.7, 29.7, 31.9, 34.3, 77.9, 172.6; ^77^Se-NMR (CDCl_3_) δ 115.0; Anal. Calcd for C_34_H_64_O_4_Se: C, 66.31; H, 10.48. Found: C, 66.34; H, 10.81.

*(±)-trans-4-Hydroxytetrahydroselenophen-3-yl heptadecanoate* (**2k**): Yield 19%. Colorless crystals. m.p. 66–68 °C. ^1^H-NMR (CDCl_3_) δ 0.87 (t, *J* = 6.9 Hz, 3H), 1.25 (m, 26H), 1.59 (m, 2H), 2.30 (t, *J* = 7.5 Hz, 2H), 2.34 (d, *J* = 6.2 Hz, 1H), 2.89 (dd, *J* = 3.0, 11.3 Hz, 1H), 2.93 (dd, *J* = 3.0, 10.7 Hz, 1H), 3.13 (dd, *J* = 4.4, 10.7 Hz, 1H), 3.22 (dd, *J* = 5.1, 11.3 Hz, 1H), 4.41 (m, 1H), 5.26 (m, 1H); ^13^C-NMR (CDCl_3_) δ 14.1, 22.7, 24.2, 24.9, 28.2, 29.1, 29.2, 29.4, 29.5, 29.6, 29.6, 29.7, 29.7, 29.7, 31.9, 34.3, 77.1, 80.2, 173.1; ^77^Se-NMR (CDCl_3_) δ 90.4; Anal. Calcd for C_21_H_40_O_3_Se: C, 60.13; H, 9.61. Found: C, 60.03; H, 9.74.

*(±)-trans-Tetrahydroselenophen-3,4-diyl diheptadecanoate* (**3k**): Yield 3%. Colorless crystals. m.p. 58–59 °C. ^1^H-NMR (CDCl_3_) δ 0.89 (t, *J* = 7.0 Hz, 6H), 1.27 (m, 52H), 1.62 (m, 4H), 2.33 (m, 4H), 2.96 (m, 2H), 3.24 (m, 2H), 5.38 (m, 2H); ^13^C-NMR (CDCl_3_) δ 14.1, 22.7, 24.3, 24.9, 29.1, 29.3, 29.4, 29.5, 29.6, 29.7, 29.7, 29.7, 30.9, 31.9, 34.3, 77.9, 172.6; ^77^Se-NMR (CDCl_3_) δ 115.0; Anal. Calcd for C_38_H_72_O_4_Se: C, 67.92; H, 10.80. Found: C, 67.93; H, 11.46.

### 3.3. Lipid Peroxidation Assay 

According to the literature method [12], a chloroform solution (10 mL) of lecithin (50 mg) and cholesterol (25 mg) was evaporated, and the residual material was dissolved in pH 7.4 phosphate buffer (50 mL) and sonicated for 20 min. The obtained liposome solution (2.1 mL each) was added with 187 μL of 0.05 M AAPH in the buffer and 0–72 μL of 5 mM antioxidant (**1**–**4**) in methanol (or acetonitrile) and diluted with the buffer to prepare 3 mL each of test solutions containing 3.1 mM AAPH and 0–120 μM antioxidant. The solutions were incubated at 37 °C for 3 h to induce lipid peroxidation. Then, the solution was added with 20 μL of 0.34 M BHT in ethanol and 3 mL of TBARS (containing 11.3 mg of TBA, 750 μL of 1 M HCl, and 1.12 mL of 40% trichloroacetic acid in H_2_O) and heated on a boiling water bath for 40 min. After centrifugation for 10 min, the UV absorption of the supernatant solution was measured at 532 nm to determine the amount of malondialdehyde (MDA) produced by lipid peroxidation as a TBA adduct. The percentage (%) inhibition of the lipid peroxidation in the presence of an antioxidant was standardized by using the absorptions observed for the blank solution, which did not contain AAPH and an antioxidant, and the reference solution, which contained AAPH but not an antioxidant. The measurement was repeated several times, and the 50% inhibitory concentration for the lipid peroxidation was obtained as an average of the three independent experiments. In the case of **2c**, however, the measurement was carried out only once because of shortage of the sample.

### 3.4. Colony Formation Assay

The assay was carried out by using the *Bacillus subtilis* subsp. *subtilis* NBRC3134 (ATCC6633) strain [21,22], which was obtained from National Institute of Technology and Evaluation (NITE) Biological Resource Center, Tokyo, Japan, following the standard method. A broth was prepared by mixing yeast (1 g), tryptone (2 g), and sodium chloride (2 g) in water (200 mL). *Bacillus subtilis* subsp. *subtilis* was added to the broth (15 mL), and the solution was incubated at 37 °C for 24 h to prepare the full-growth state containing 1 × 10^8^ cells per 1 mL broth. The resulting solution was diluted 10^5^ times with the broth. The test compound (**1**, **2h** or **4**) dissolved in methanol was added as a portion to 10 mL each of the diluted solution. A blank solution (without the test compound) and a series of assay solutions with varying concentration of the test compound were prepared. They were subsequently incubated at 37 °C for 24 h. In every two hours, an aliquot of the solution (10 μL) was taken, and the colony forming units were counted by dilution, inoculation on the partitioned agar petri dishes, and incubation at 37 °C for 24 h. The agar dishes were prepared from the broth (200 mL) and agar (3 g).

## 4. Conclusions

We designed in this study DHS-fatty acid conjugates **2a**–**l** having a variable alkyl chain length and assayed their antioxidant capacities to inhibit lipid peroxidation by using a lecithin/cholesterol liposome. As a result, monoesters **2f**–**j** with a long alkyl chain showed high antioxidant activities against accumulation of LOOH. On the other hand, monoesters with a short alkyl chain (compounds **2a** and **2b**), showed lower activities than parent **1**. Tridecanoic acid diester (**3g**) showed the similar activity to **1**. Myristic acid monoester (**2h**), one having a high activity (IC_50_ = 18 μM), was then employed for the colony formation assay using *Bacillus subtilis* to evaluate its interaction to living cell membranes. The bacterial cell multiplication was inhibited by **2h** at the concentration larger than 160 μM, suggesting its ability to interact with the cell membrane. These observations were consistent with the antioxidant function previously proposed for **2** [12]. Similarity of the antioxidant functions between **2** and GPx4 (Scheme 3) suggested that fatty acid monoesters of DHS can mimic the LOOH degradation function of GPx4. In conclusion, importance of the balance between hydrophilicity and lipophilicity for the design of effective GPx4 mimics was suggested. More effective AOs that mimic the function of GPx4 in human cellular environments, are being explored in our laboratories.

## Supplementary Materials

Supplementary materials can be accessed at: http://www.mdpi.com/1420-3049/20/07/12364/s1.

## References

[1] Margis R., Dunand C., Teixeira F.K., Margis-Pinheiro M. (2008). Glutathione peroxidase family—An evolutionary overview. FEBS J..

[2] Brigelius-Flohé R., Maiorino M. (2013). Glutathione peroxidases. Biochim. Biophys. Acta.

[3] Ursini F., Heim S., Kiess M., Maiorino M., Roveri A., Wissing J., Flohé L. (1999). Dual function of the selenoprotein PHGPx during sperm maturation. Science.

[4] Ufer C., Wang C.C., Fähling M., Schiebel H., Thiele B.J., Billett E.E., Kuhn H., Borchert A. (2008). Translational regulation of glutathione peroxidase 4 expression through guanine-rich sequence-binding factor 1 is essential for embryonic brain development. Genes Dev..

[5] Schneider M., Förster H., Boersma A., Seiler A., Wehnes H., Sinowatz F., Neumüller C., Deutsch M.J., Walch A., Hrabé de Angelis M. (2009). Mitochondrial glutathione peroxidase 4 disruption causes male infertility. FASEB J..

[6] Mugesh G., Singh H.B. (2000). Synthetic organoselenium compounds as antioxidants: glutathione peroxidase activity. Chem. Soc. Rev..

[7] Mugesh G., du Mont W.W., Sies H. (2001). Chemistry of biologically important synthetic organoselenium compounds. Chem. Rev..

[8] Bhabak K.P., Mugesh G. (2010). Functional mimics of glutathione peroxidase: Bioinspired synthetic antioxidants. Acc. Chem. Res..

[9] Raneva V., Shimasaki H., Furukawa Y., Ueta N., Yanishlieva N., Aaseng J.E., Partali V., Sliwka H.R., Yoshida Y., Niki E. (2002). Action of 1-(11-selenadodecyl)-glycerol and 1-(11-selenadodecyl)-3-trolox-glycerol against lipid peroxidation. Lipids.

[10] Shanks D., Amorati R., Fumo M.G., Pedulli G.F., Valgimigli L., Engman L. (2006). Synthesis and antioxidant profile of all-rac-α-selenotocopherol. J. Org. Chem..

[11] Kumakura F., Mishra B., Priyadarsini K.I., Iwaoka M. (2010). A water-soluble cyclic selenide with enhanced glutathione peroxidase-like catalytic activities. Eur. J. Org. Chem..

[12] Iwaoka M., Sano N., Lin Y.Y., Katakura A., Noguchi M., Takahashi K., Kumakura F., Arai K., Singh B.G., Kunwar A. (2015). Fatty acid conjugates of water-soluble (±)-*trans*-selenolane-3,4-diol: Effects of alkyl chain length on the antioxidant capacity. ChemBioChem.

[13] Phadnis P.P., Wadawale A., Priyadarsini K.I., Jain V.K., Iwaoka M. (2015). Synthesis, characterization, and structure of *trans*-3,4-dihydroxy-1-selenolane {DHS(OH)_2_} substituted derivatives. Tetrahedron Lett.

[14] Iwaoka M., Takahashi T., Tomoda S. (2001). Syntheses and structural characterization of water-soluble selenium reagents for the redox control of protein disulfide bonds. Heteroat. Chem..

[15] Meotti F.C., Stangherlin E.C., Zeni G., Nogueira C.W., Rocha J.B.T. (2004). Protective role of aryl and alkyl diselenides on lipid peroxidation. Environ. Res..

[16] Santos F.W., Zeni G., Rocha J.B.T., Weis S.N., Fachinetto J.M., M. Favero A., Nogueira C.W. (2005). Diphenyl diselenide reverses cadmium-induced oxidative damage on mice tissues. Chem. Biol. Interact..

[17] De Freitas A.S., de Souza Prestes A., Wagner C., Sudati J.H., Alves D., Porciúncula L.O., Kade I.J., Rocha J.B.T. (2010). Reduction of diphenyl diselenide and analogs by mammalian thioredoxin reductase is independent of their gluthathione peroxidase-like activity: A possible novel pathway for their antioxidant activity. Molecules.

[18] Da Rocha J.T., Pinton S., Mazzanti A., Mazzanti C.M., Beckemann D.V., Nogueira C.W., Zeni G. (2011). Effects of diphenyl diselenide on lipid profile and hepatic oxidative stress parameters in ovariectomized female rats. J. Pharm. Pharmacol..

[19] Ohkawa H., Ohishi N., Yagi K. (1979). Assay for lipid peroxides in animal tissues by thiobarbituric acid reaction. Anal. Biochem..

[20] Cai Y.J., Fang J.G., Ma L.P., Yang L., Liu Z.L. (2003). Inhibition of free radical-induced peroxidation of rat liver microsomes by resveratrol and its analogues. Biochim. Biophys. Acta.

[21] Michener H.D. (1955). The action of subtilin on heated bacterial spores. J. Bacteriol..

[22] Sakamoto T., Hours R.A., Sakai T. (1994). Purification, characterization, and production of two pectic transeliminases with protopectinase activity from *Bacillus subtilis*. Biosci. Biotech. Biochem..

[23] Auclair C., Voisin E., Banoun H., Paoletti C., Bernadou J., Meunier B. (1984). Potential antitumor agents: Synthesis and biological properties of aliphatic amino acid 9-hydroxyellipticinium derivatives. J. Med. Chem..

[24] Russell A.D. (1990). Bacterial spores and chemical sporicidal agents. Clin. Microbiol. Rev..

[25] Arai K., Dedachi K., Iwaoka M. (2011). Rapid and quantitative disulfide bond formation for a polypeptide chain using a cyclic selenoxide reagent in an aqueous medium. Chem. Eur. J..

