# Supplementary material for: Mimicking the Lipid Peroxidation Inhibitory Activity of Phospholipid Hydroperoxide Glutathione Peroxidase (GPx4) by Using Fatty Acid Conjugates of a Water-Soluble Selenolane
Source: Molecules. 2015 Jul 7;20(7):12364–75. doi: 10.3390/molecules200712364 (PMC6331923; doi:10.3390/molecules200712364)
Supplement: Supplementary file 1 [file molecules-20-12364-s001.pdf]

Supplementary Materials

1. Data of Lipid Peroxidation Experiments

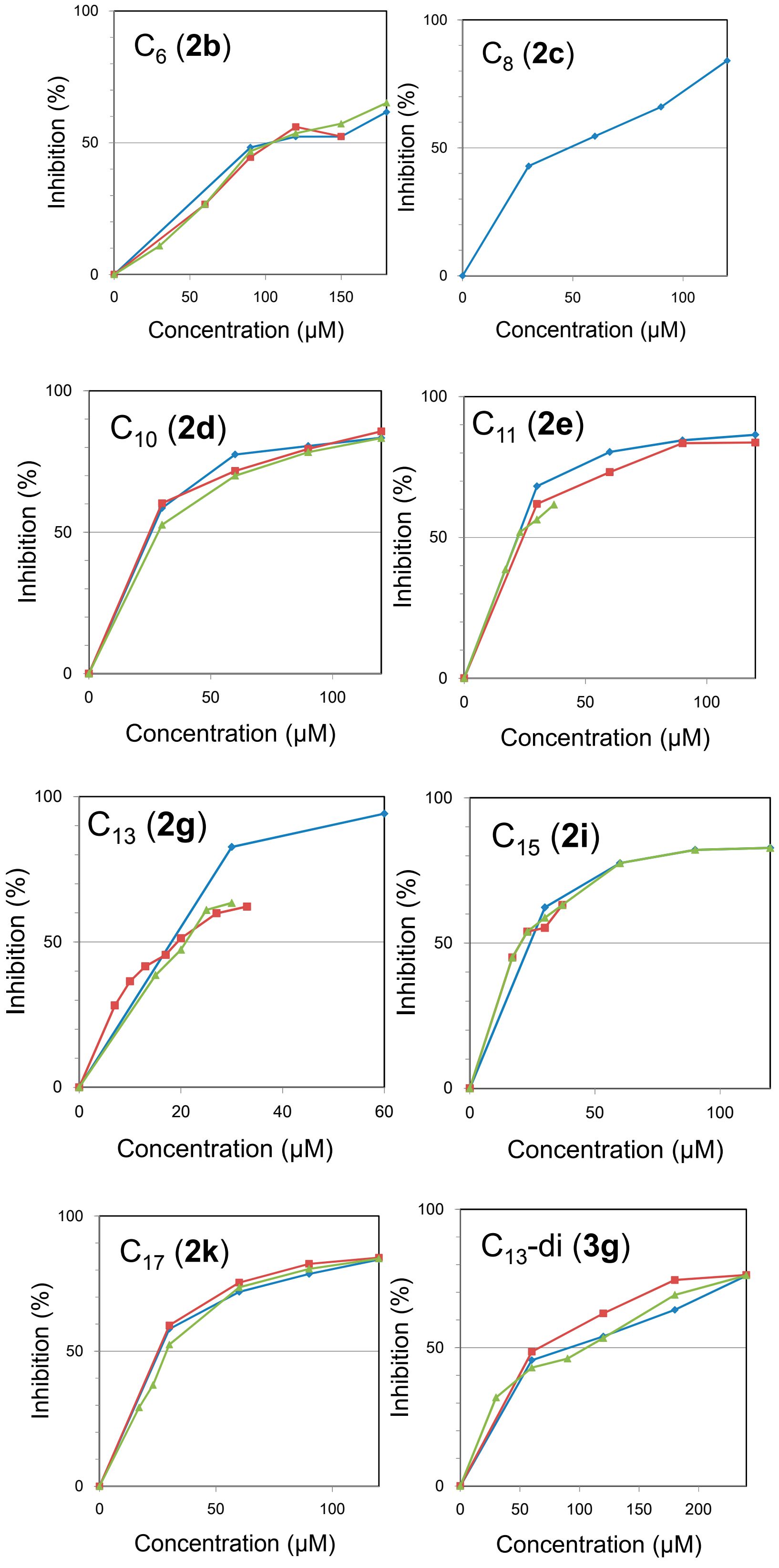

Figure S1. Inhibition ratio of lipid peroxidation in the lecithin-cholesterol liposome assay.

Table S1. 50% inhibitory concentrations (IC_{50}) against lipid peroxidation obtained for **1–4** [a].

Compounds	IC_{50} (μM)	Compounds	IC_{50} (μM)	Compounds	IC_{50} (μM)
DHS (**1**)	64 ±5 [b]	C_{11} (**2e**)	23 ± 2	C_{16} (**2j**)	25 ± 6 [b]
C_3 (**2a**)	87 ± 2 [b]	C_{12} (**2f**)	23 ± 4 [b]	C_{17} (**2k**)	27 ± 2
C_6 (**2b**)	104 ± 1	C_{13} (**2g**)	19 ± 2	C_{18} (**2l**)	34 ± 3 [b]
C_8 (**2c**)	48 [c]	C_{14} (**2h**)	18 ± 1 [b]	C_{13}-di (**3g**)	80 ± 13
C_{10} (**2d**)	26 ± 2	C_{15} (**2i**)	22 ± 3	PhSeSePh (**4**)	70 ± 4 [b]

[a] Errors are given as standard deviations. [b] Data are quoted from Ref. 12. [c] Measurement was carried out only once.

2. Data of Colony Formation Assays

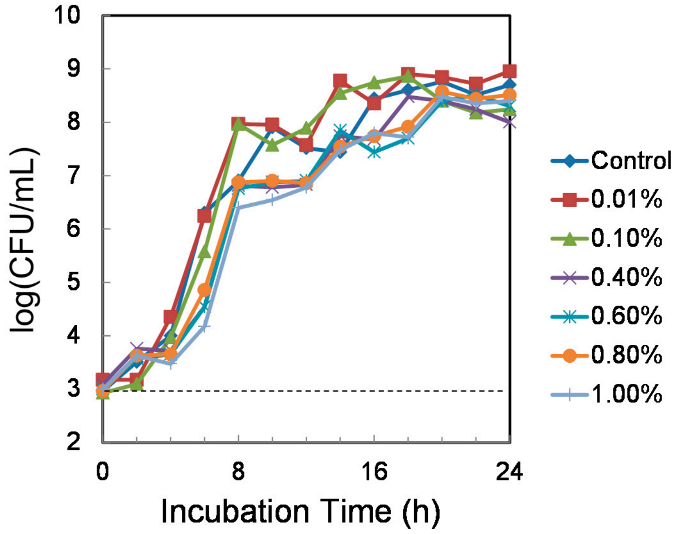

Figure S2. The number of colony forming units (CFU) of NBRC3134 as a function of the incubation time in the presence of DHS (**1**).

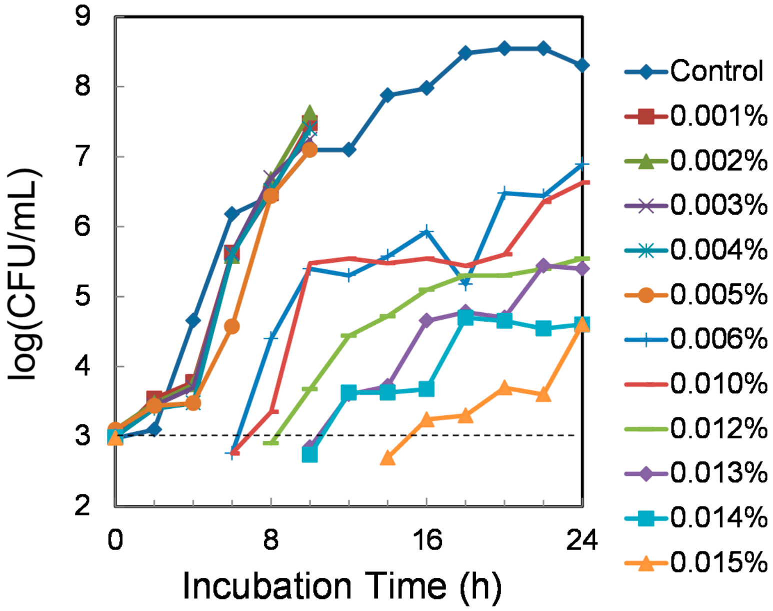

Figure S3. The number of colony forming units (CFU) of NBRC3134 as a function of the incubation time in the presence of C_{14} (**2h**).

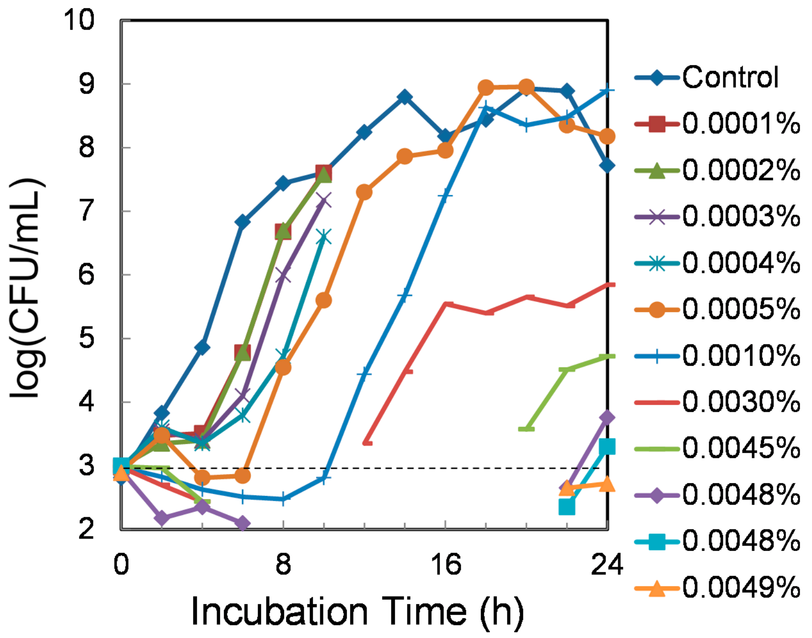

Figure S4. The number of colony forming units (CFU) of NBRC3134 as a function of the incubation time in the presence of PhSeSePh (**4**).

3. ^{1}H-, ^{13}C-, and ^{77}Se-NMR Spectra for 3b

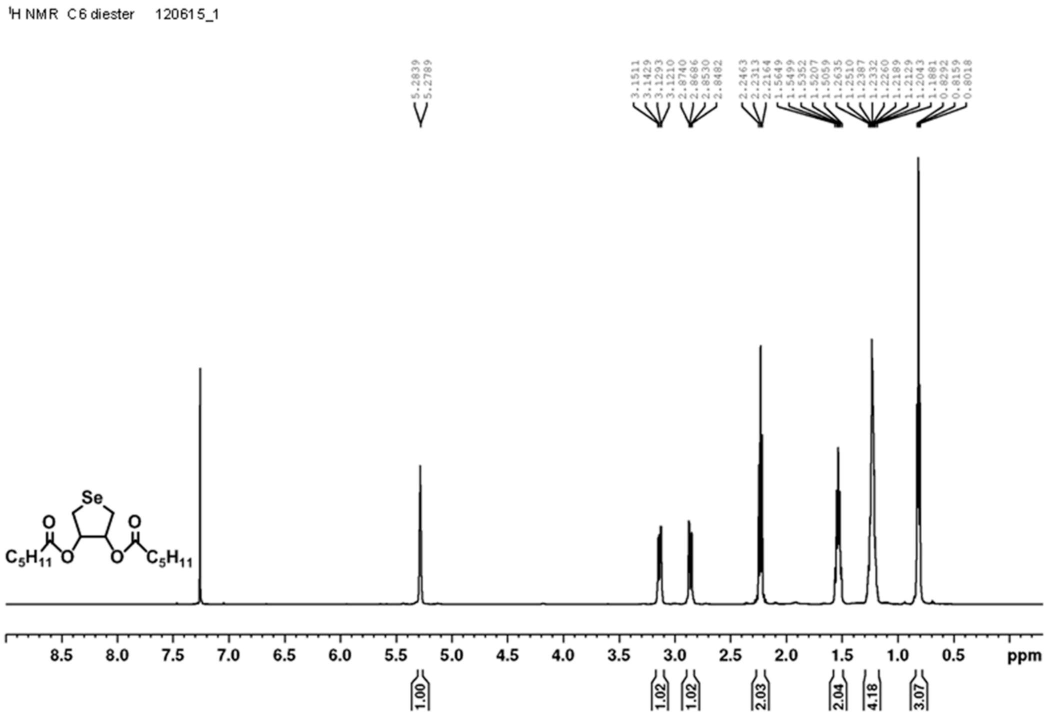

Figure S5. ^{1}H-NMR Spectra for **3b**.

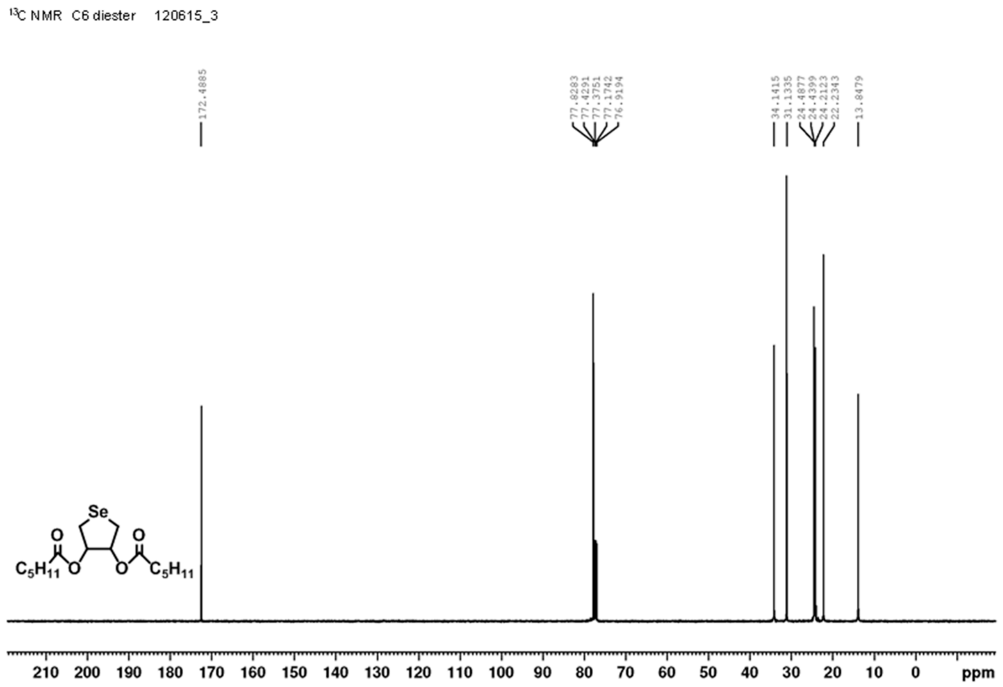

Figure S6. ^{13}C-NMR Spectra for **3b**.

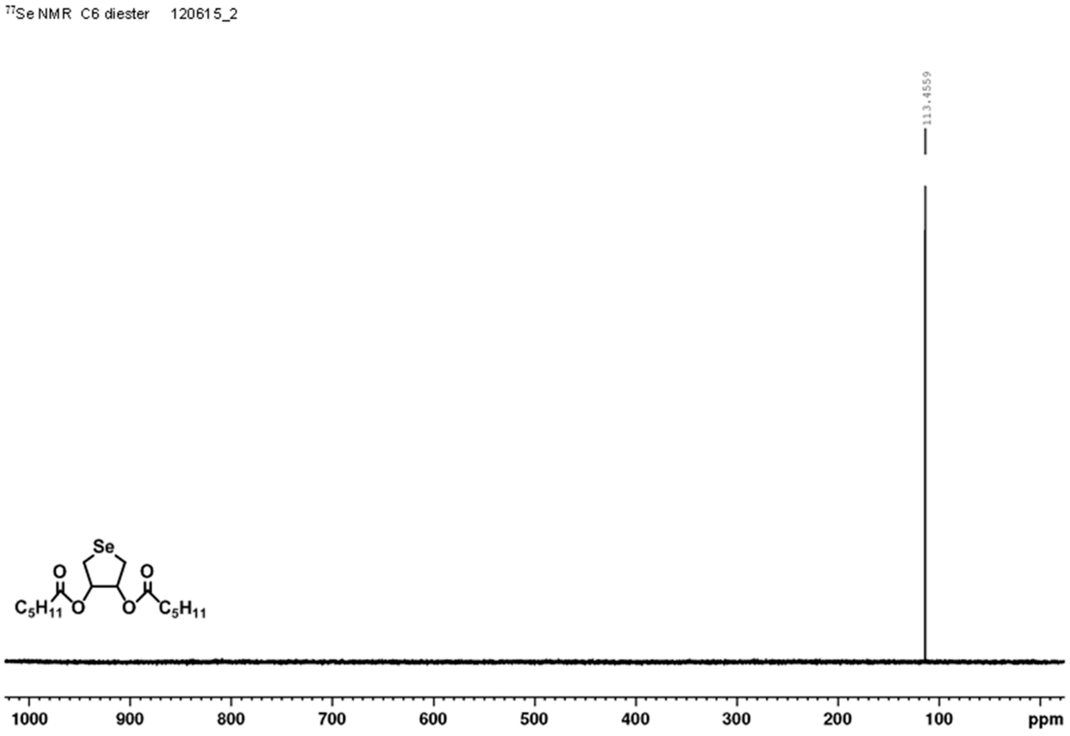

Figure S7. ^{77}Se-NMR Spectra for **3b**.
